# A Novel MEMS Capacitive Microphone with Semiconstrained Diaphragm Supported with Center and Peripheral Backplate Protrusions

**DOI:** 10.3390/mi13010022

**Published:** 2021-12-25

**Authors:** Shubham Shubham, Yoonho Seo, Vahid Naderyan, Xin Song, Anthony J. Frank, Jeremy Thomas Morley Greenham Johnson, Mark da Silva, Michael Pedersen

**Affiliations:** Knowles Corporation, LLC., Itasca, IL 60143, USA; yoonho.seo@knowles.com (Y.S.); vahid.naderyan@knowles.com (V.N.); xin.song@knowles.com (X.S.); tony.frank@knowles.com (A.J.F.II); jeremy.johnson@knowles.com (J.T.M.G.J.); mark.dasilva@knowles.com (M.d.S.); michael.pedersen@knowles.com (M.P.)

**Keywords:** MEMS, capacitive microphone, finite element modeling, reduced order modeling, effective area, peripheral protrusion, center protrusion, serpentine spring

## Abstract

Audio applications such as mobile phones, hearing aids, true wireless stereo earphones, and Internet of Things devices demand small size, high performance, and reduced cost. Microelectromechanical system (MEMS) capacitive microphones fulfill these requirements with improved reliability and specifications related to sensitivity, signal-to-noise ratio (SNR), distortion, and dynamic range when compared to their electret condenser microphone counterparts. We present the design and modeling of a semiconstrained polysilicon diaphragm with flexible springs that are simply supported under bias voltage with a center and eight peripheral protrusions extending from the backplate. The flexible springs attached to the diaphragm reduce the residual film stress effect more effectively compared to constrained diaphragms. The center and peripheral protrusions from the backplate further increase the effective area, linearity, and sensitivity of the diaphragm when the diaphragm engages with these protrusions under an applied bias voltage. Finite element modeling approaches have been implemented to estimate deflection, compliance, and resonance. We report an 85% increase in the effective area of the diaphragm in this configuration with respect to a constrained diaphragm and a 48% increase with respect to a simply supported diaphragm without the center protrusion. Under the applied bias, the effective area further increases by an additional 15% as compared to the unbiased diaphragm effective area. A lumped element model has been also developed to predict the mechanical and electrical behavior of the microphone. With an applied bias, the microphone has a sensitivity of −38 dB (ref. 1 V/Pa at 1 kHz) and an SNR of 67 dBA measured in a 3.25 mm × 1.9 mm × 0.9 mm package including an analog ASIC.

## 1. Introduction

After the commercialization of the first microelectromechanical system (MEMS) microphone by Knowles in 2002 [1], the microphone market has witnessed a giant leap toward high-performance audio applications meant for consumer electronics, automotive, hearing aids, military, and aerospace markets. The capacitive MEMS microphone industry has attracted significant interest because these designs facilitate small size, reduced cost, low power consumption, high sensitivity, low noise, flat frequency response, and good stability with respect to temperature and humidity [2,3]. The silicon micromachining technology enables high-volume production of MEMS microphone devices with an outstanding level of miniaturization that can be achieved within an area less than 1 mm^2^ [4]. Alternate transduction mechanisms, i.e., piezoelectric [5], piezoresistive [6,7], and optical [8,9], have also been demonstrated to convert the sound pressure waves into the electrical signal.

In order to design a MEMS microphone and predict its behavior, simplified network modeling is typically used due to the complex nature of the coupled acoustical, mechanical, and electrical components. Extensive network modeling approaches have been discussed over the past years and have been published [10,11]. To improve the sensitivity, a diaphragm having slits at the edges was proposed by Yoo et al. [12]. Circular diaphragms with and without slits at the edges of the diaphragm have been investigated, in which the diaphragm with slits shows a higher displacement. A deflected membrane causes a change in the effective area of the diaphragm, and the capacitance is decreased, causing lower sensitivity. In order to overcome this problem, the authors in [13] suggested two coupled membranes in which the first membrane closer to the port experiences the sound pressure and the other membrane attached at the center of the first membrane acts as a moving electrode.

Thin-film diaphragms usually are affected by residual stresses of the film, which can cause issues related to high diaphragm deflection and, even in some cases, buckling of the diaphragm after the release process. The mechanical compliance of the membranes is further limited by the residual stresses in the released layer, potentially altering sensitivity. Different techniques have been used in the past to reduce the residual stresses in the thin films, such as corrugations in the diaphragms, slits on the diaphragm, spring-supported structures, and controlling the deposition parameters in the fabrication process. With the increase in the number of corrugations in the diaphragm, the mechanical sensitivity can be increased [14]. However, this trend is not always observed to be true, as the mechanical sensitivity tends to reduce after a certain number of corrugations as it begins to stiffen up the diaphragm further to acoustic level pressure loads. A strong function of corrugation width with number of corrugations is discussed in [15]. In [16], finite element analysis was used to optimize the number of corrugations and corrugation depth. The diaphragm with different types of springs was analyzed analytically, and FEA simulations were performed to check and compare their sensitivities. In [17], a frog-arm-supported diaphragm is presented, which shows higher sensitivities as compared to that with a simple clamped diaphragm. Sui et al. [18] analyze how a spring-supported diaphragm can achieve improved sensitivity with a reduction in diaphragm radius as compared to the simple clamped design [18]. Instead of using a flexible diaphragm, a microphone consisting of a rigid diaphragm supported by flexible springs is proposed [19], which helped reduce the diaphragm deformation caused by thin-film residual stress. This design was further improved in terms of sensitivity, signal-to-noise ratio (SNR), and bandwidth by using a flexible V-shaped spring, silicon nitride electrical isolation, and the ring-type oxide/Polysilicon mesa, respectively [20].

Other research studies (on dual backplate designs) have also shown advantages in sensitivity and SNR compared to a single-backplate microphone [21,22,23,24]. SNR, a key indicator of performance, is the most important parameter of MEMS microphones and is defined as the difference between a microphone’s sensitivity and its noise floor and is expressed in decibels (dB, A-weighted). Current MEMS microphones with analog application-specific integrated circuits (ASIC) can be found in the range of about 60 dB to 73 dB SNR [25] by balancing high sensitivity and low self-noise of the microphone. Improving the SNR can also be achieved by designing a fully differential architecture double-diaphragm [26] or double-backplate microphone with performance benefits [27].

In this paper, we will focus on improving the SNR by increasing the effective area of the diaphragm with peripheral and center protrusions on the backplate acting like a simply supported boundary condition for the diaphragm. The polysilicon diaphragm is suspended using serpentine springs to minimize the residual film stress of the diaphragm. In addition, analytical, finite element, and lumped element models are established to predict the electroacoustic behavior of the proposed microphone whose performance is in good agreement with the measurements.

## 2. Microphone Structure and Design

**Figure 1:**
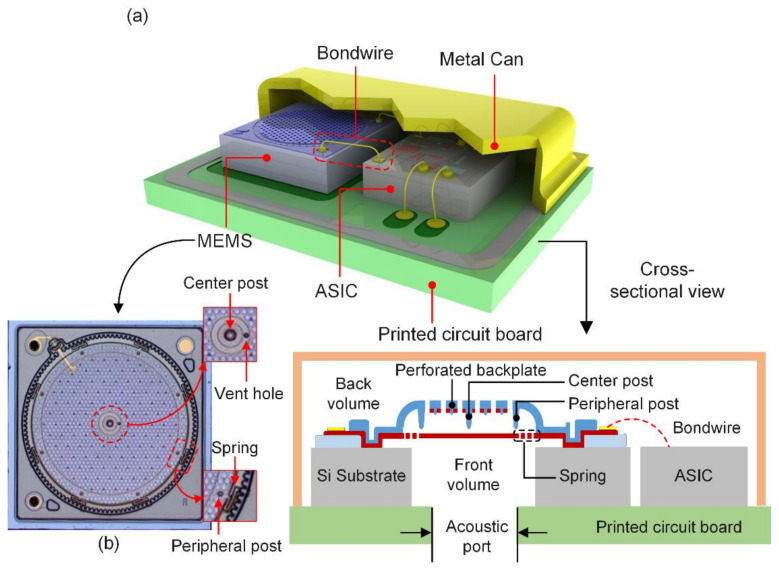
(**a**) A schematic cross-sectional view of a generic Knowles MEMS microphone package. (**b**) A top–down optical image of the MEMS die collected using a microscope. The movable diaphragm is suspended with eight compliant springs along the perimeter. The center and eight peripheral posts on the backplate prevent the movable diaphragm from electrostatic collapse onto the backplate.

A generic Knowles capacitive MEMS microphone is represented in Figure 1a. The cross-section of the structure consists of a polysilicon diaphragm suspended on springs that are constrained at the perimeter region, as shown in Figure 1b. When a bias voltage is applied, the diaphragm is electrostatically attracted to the backplate but simply supported with a center and eight peripheral protrusions (or posts) that extend from the backplate. A variable capacitor is formed by the relatively rigid backplate and the movable diaphragm. In response to acoustic pressure changes, the capacitance of the diaphragm changes, and this change is sensed and converted into a proportional electrical voltage through the ASIC.

An optical microscope image of the MEMS die is shown in Figure 1b. It clearly shows the eight serpentine spring structures of the diaphragm which are aligned with the peripheral posts. The serpentine springs are anchored with the perimeter anchor structure of the backplate. A small vent hole is located near the center post to allow ventilation of the pressure between the front and back volume of the microphone. This is used to control the low-frequency roll-off (LFRO) by forming an acoustical high pass filter for sound. The bias voltage is applied to the diaphragm bond pad, and the change in capacitance as output signal is measured across the backplate bond pad.

**Figure 2:**
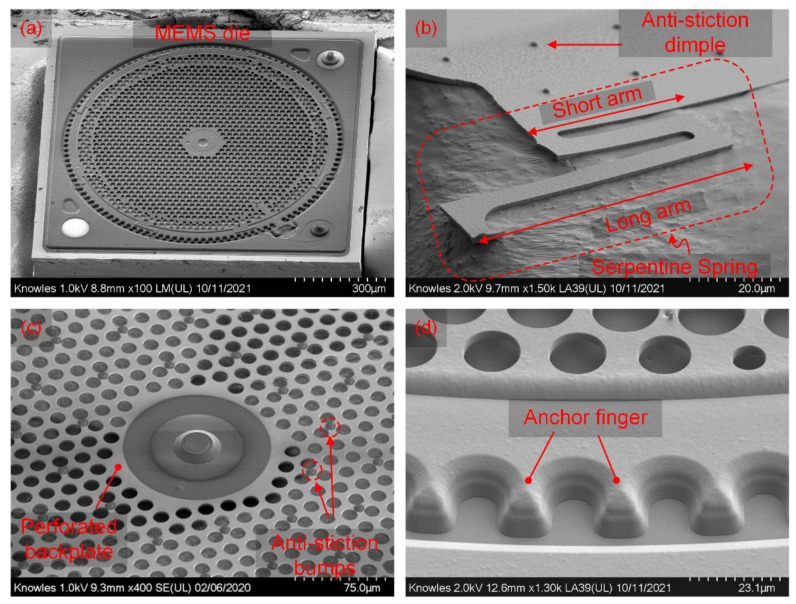
SEM images of critical features of the proposed design. (**a**) Top view of the MEMS microphone die, (**b**) serpentine spring design, (**c**) perforated backplate with antistiction bumps, and (**d**) backplate anchor design to substrate.

Details of the MEMS structure are visible in Figure 2a–d. Figure 2a shows the top–down view of the MEMS die. Figure 2b is a close-up view of the serpentine spring that comprises spring arms with different lengths. The long arm connected to anchor fingers, as shown in Figure 2b, provides enough mechanical compliance to achieve the desired high sensitivity, whereas the short arm connected to the diaphragm provides torsional rigidity to the diaphragm and more uniform lift-off under bias voltage. Figure 2b also shows the antistiction dimple structure present underneath the diaphragm.

Figure 2c presents perforation holes formed in the backplate. Figure 2d shows the backplate anchor design to the substrate. 

## 3. Microfabrication

**Figure 3:**
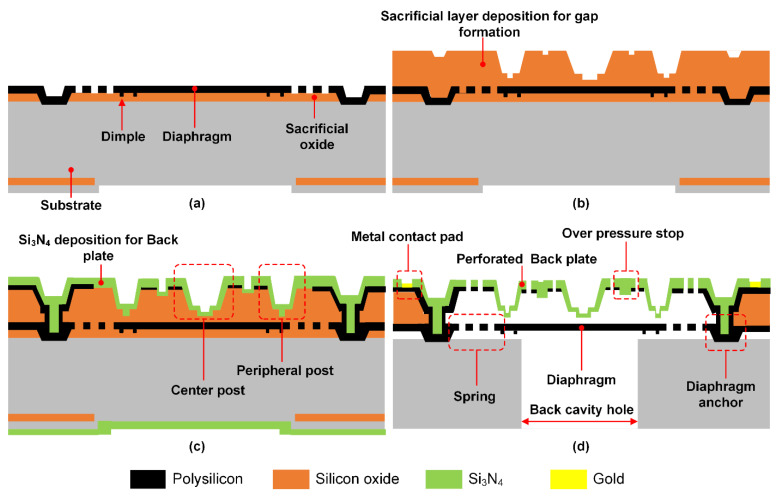
Proposed design fabrication sequence: (**a**) 1st sacrificial layer deposition and polysilicon deposition for diaphragm with dimples. (**b**) Sensing gap formation with sacrificial layer deposition and patterning. (**c**) Si_3_N_4_ deposition for formation of backplate with polysilicon electrode with a center and peripheral protrusions. (**d**) Backplate patterning, formation of the back cavity, and gold metal pads with sacrificial layer release.

The schematic in Figure 3 shows a simplified MEMS fabrication process for the MEMS device. The first sacrificial layer deposition is followed by polysilicon diaphragm deposition and etch (Figure 3a). The next sacrificial layer deposition will form a gap between the diaphragm and backplate electrode and also define the shape of the peripheral and center posts (Figure 3b). After that, the polysilicon is deposited and patterned to form an electrode for a backplate formed by the subsequent Si_3_N_4_ deposition (Figure 3c). The backplate is patterned to form acoustic holes, and gold is deposited to form metal contact pads. The back cavity hole is formed by a silicon deep-reactive ion etch process. Then, the sacrificial oxide is removed (Figure 3d). 

## 4. Reduced Order Modeling

**Figure 4:**
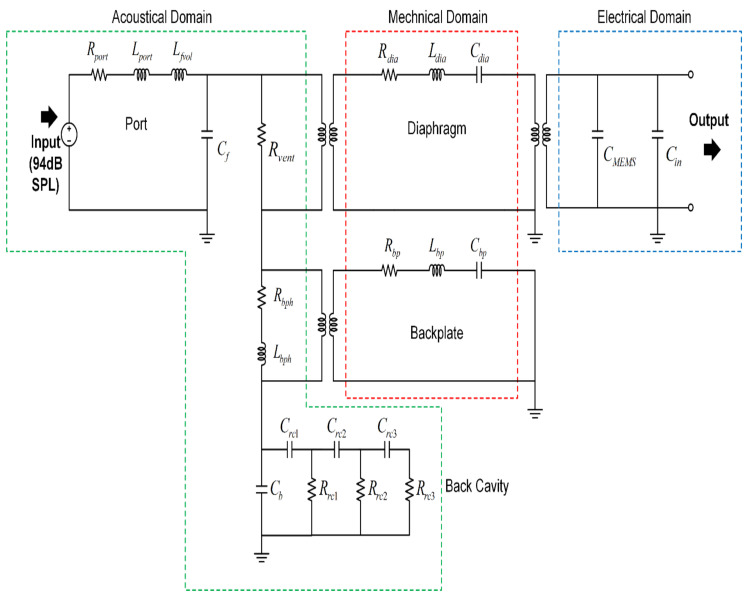
A reduced order model for a single-motor MEMS microphone.

A proposed reduced order model for the MEMS microphone is shown in Figure 4. The diaphragm mechanical model is coupled with acoustic and electrical domains on its left- and right-hand side, respectively. This coupling is represented using transformers, which account for flow and effort variables in each domain, as shown in Table 1. The flow of air or sound pressure represented in the acoustic domain creates mechanical movement of the diaphragm, which further drives the electric circuit by changing the capacitance between the motor and electrode.

In terms of the elements such as mass, spring, and damper, the lumped equivalent mechanical, electrical, and acoustical models are represented in Table 2.

The description of each element for the proposed reduced order model is presented in Table 3.

The acoustical lumped parameters are related to mechanical lumped parameters using the Equation (1):

(1) $$Z_{a}=Z_{m}/A_{d\_eff}{}^{2}$$

where $Z_{m}\;\mathrm{is}\;\mathrm{the}\;\mathrm{mechanical}\;\mathrm{impedance},\;Z_{a}\;\mathrm{is}\;\mathrm{the}\;\mathrm{acoustical}\;\mathrm{impedance},\;\mathrm{and}\;A_{d\_eff}$ is the effective diaphragm area.

### 4.1. Diaphragm Effective Area (Acoustic-to-Mechanical Transformer Ratio)

**Figure 5:**
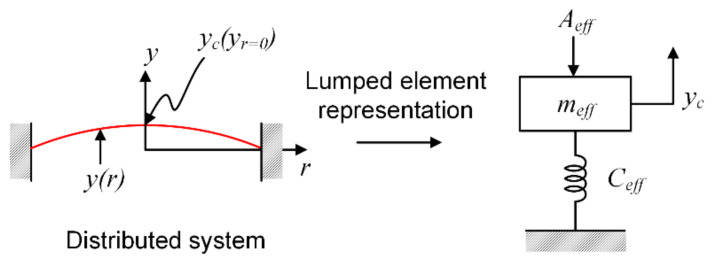
Lumped element representation of a distributed system.

The transformer that turns the ratio for conversion between the acoustical and mechanical domains is the diaphragm area. An effective area is typically used instead of a geometric one to better represent a distributed system as the network of lumped elements, as shown in Figure 5. The effective area is defined so that the air volume displaced by the lumped element system is equal to the air volume displaced by the distributed system. 

Mathematically it can be stated as,

(2) $$A_{d\_eff}\cdot y_{c}=\int_{0}^{a}y\left(r\right)2\pi rdr$$

where the left side of the Equation (2) is the volume displaced by the lumped piston model, and the right side of the Equation (2) is the volume displaced by the distributed system, where $y_{c}$ is the maximum deflection of the diaphragm, a is the diaphragm radius, and $y\left(r\right)$ is the diaphragm deflection as a function of radius. 

The Equation (2) can be rearranged to define a lumped area coefficient so that

(3) $$A_{d\_eff}=\beta A_{d}$$

where $A_{d}$ is the geometric area of the diaphragm.

The effective area coefficient is defined as

(4) $$\beta =\frac{A_{d\_eff}}{A_{d}}=\frac{\int_{0}^{a}y\left(r\right)2\pi rdr}{A_{d}y_{c}}$$

The effective area coefficient, β, can be obtained by integrating analytical equations for diaphragm displacement or by numerical integration of actual displacement data for the diaphragm.

To determine β, one must know $y\left(r\right)$, the deflected shape of the diaphragm. In this section, we use analytical equations for clamped and simply supported plates to obtain β for each case and then compare the results to ones obtained using FEA.

The expression for obtaining the small deflection shape of a simply supported boundary condition is given by [28]:

(5) $$y\left(r\right)=\frac{q\left(a^{2}-r^{2}\right)}{64D}\left(\left(\frac{5+\nu }{1+\nu }\right)a^{2}-r^{2}\right)$$

where q is the uniform pressure difference across the diaphragm, D is the flexural rigidity for the diaphragm as determined by material properties and diaphragm dimensions, and v is the Poisson’s ratio.

However, the constrained diaphragm or clamped boundary condition with no residual stress is given by [28]:

(6) $$y\left(r\right)=\frac{q}{64D}{\left(a^{2}-r^{2}\right)}^{2}$$

Substituting Equations (5) and (6) above into the Equation (4) for β and solving the integral yields:

β=0.46 for a simply supported plate and $\beta =\frac{1}{3}\approx 0.33$ for a constrained diaphragm. Both calculations are for a generic polysilicon diaphragm with assumed elastic properties.

**Figure 6:**
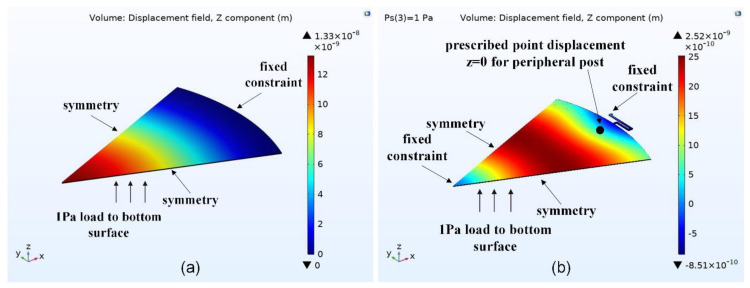
Defined boundary conditions and z−displacement field for: (**a**) constrained diaphragm and (**b**) spring−supported diaphragm with peripheral and center post under bias.

An FEA approach has been used to verify these analytical results and the effective area coefficient, *β*, for peripheral posts and center post boundary conditions for free plate diaphragm without springs and a semiconstrained diaphragm with springs. Results of the FEA simulations are shown in Figure 6 and summarized in Table 4.

The FEA is set up with a 45-degree symmetric boundary condition, and a small signal load of 1Pa pressure is applied to make the diaphragm deflect. The effective area coefficient, β, is calculated based on Equation (4), defined above.

The diaphragm area, $A_{d}$, is calculated to be 5.671 $\times 10^{-7}$ $m^{2}$. For a perfect piston-shaped diaphragm deflection, *β* is ~1. The higher β is, the closer deflection of the diaphragm is to the piston-shaped motion. The diaphragm with the peripheral and center post configuration has an 85% increase in the effective area as compared to the constrained diaphragm. The center post configuration combined with the peripheral posts has a 48% higher effective area compared to the peripheral post configuration without any center post. Under an applied bias voltage, the effective area additionally increases by 15% as compared to unbiased case for the proposed configuration since the diaphragm is more stiffened under bias voltage. This increase is also observed in the case of free plate with peripheral and center post.

### 4.2. Electrostatic Coupling Coefficient (Mechanical-to-Electrical Transformer Ratio)

The electrostatic coupling coefficient, φ, can be calculated directly from the equations governing ideal transformers and the definitions of the effort and flow variables. Assuming the diaphragm is blocked so that it cannot move, the electrostatic force can be expressed as

(7) $$F=\varphi V_{ac}=\frac{\varepsilon AV^{2}}{2g_{o}^{2}}$$

where expression on the right-hand side is the force between the plates of a parallel plate capacitor. Note that $V^{2}$ consists of both a DC component (the bias voltage) and an AC component (the signal). Because we are interested in a linear, small-signal model, the $V_{bias}^{2}$ (DC) and $V_{ac}^{2}$ (nonlinear) terms can be dropped, and the expression becomes

(8) $$F=\varphi V_{ac}=\frac{\varepsilon A\left(V_{bias}^{2}+V_{ac}^{2}+2V_{bias}V_{ac}\right)}{2g_{o}^{2}}\approx \frac{\varepsilon AV_{bias}V_{ac}}{g_{o}^{2}}$$

Solving for the coupling coefficient yields

(9) $$\varphi =\frac{\varepsilon AV_{bias}}{g_{o}^{2}}=\frac{C_{o}}{C_{em}}$$

where $C_{o}$ is the MEMS capacitance at the bias voltage, and $C_{em}=\frac{g_{o}}{V_{bias}}$ is the reactance of the transducer [29], where $g_{o}$ is the gap after bias.

### 4.3. Effective Mass of the Diaphragm

**Figure 7:**
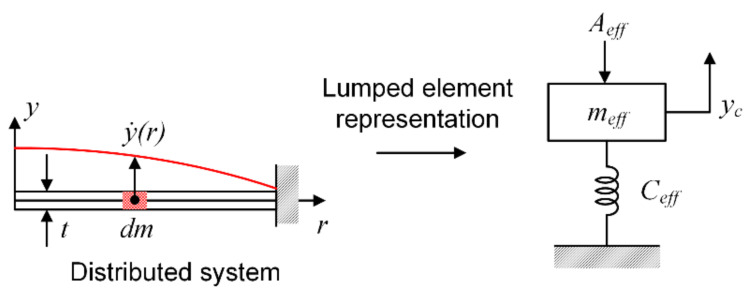
Lumped element representation of a system with distributed mass.

The effective mass of the diaphragm accounts for the nonuniform deflection profile of the diaphragm (not all of the mass is moving by the same amount). Referring to Figure 7, the effective mass used in the lumped model is defined such that kinetic energy is equal for the lumped and distributed representations. The effective mass can be expressed as

(10) $$m_{d\_eff}=\frac{2\pi \rho t}{y_{c}{}^{2}}\int_{0}^{a}y^{2}\left(r\right)rdr$$

An effective mass coefficient, α, can be defined such that $m_{d\_eff}=\alpha m_{d}$, which results in

(11) $$\alpha =\frac{m_{d\_eff}}{m_{d}}=\frac{2\pi \rho t}{m_{d}y_{c}{}^{2}}\int_{0}^{a}y^{2}\left(r\right)rdr$$

The values of α for free plate and clamped diaphragms are calculated for each case as shown below. Analytical expressions for $y\left(r\right)$ are given in Equations (5) and (6) for simply supported and clamped boundary conditions, respectively. Substituting each into the Equation (13) for the effective mass coefficient and solving yields, $\alpha =\frac{m_{d\_eff}}{m_{d}}=\frac{2\pi \rho t}{m_{d}y_{c}{}^{2}}\int_{0}^{a}y^{2}\left(r\right)rdr=0.296$ for a simply supported plate, and $\alpha =\frac{m_{d\_eff}}{m_{d}}=\frac{2\pi \rho t}{m_{d}y_{c}{}^{2}}\int_{0}^{a}y^{2}\left(r\right)rdr=0.2$ for a clamped plate.

Another technique that can be used to extract the effective mass is by measuring the resonance frequency in vacuum, which accounts for the unloaded diaphragm resonance. The effective mass equation is given by

(12) $$m_{d,eff}=\frac{1}{\omega_{o,vac}^{2}C_{d,mech}}=\frac{A_{d,eff}}{\omega_{o,vac}^{2}C_{d,cp}}$$

where $\omega_{o,vac}$ is the measured resonance frequency in vacuum and $C_{d,mech}$ is the mechanical compliance of the diaphragm in units of m/N. The mechanical compliance of the diaphragm can be also expressed in terms of effective area, $A_{d,eff}$, of the diaphragm and acoustic compliance, $C_{d,\;cp}$, in units of m/Pa. This technique is used for direct measurements for the semiconstrained MEMS with peripheral posts and center post boundary conditions.

### 4.4. Diaphragm Compliance

The diaphragm compliance is measured under an applied bias voltage. There is often a negative mechanical compliance included in the lumped models for electrostatic transducers that accounts for a phenomenon commonly referred to as electrostatic softening, whereby the mechanical compliance is effectively increased by the tendency of the electrostatic force to counteract the diaphragm restoring force when the diaphragm is displaced toward the counter electrode [29]. However, the negative compliance is not included in the model presented here, since the MEMS is biased when the compliance is measured. The softening term is already accounted for during the measurement, and no separate term is needed in the model. The compliance values obtained are only valid for the bias at which they are measured. If the bias is changed, the compliance needs to be remeasured.

**Figure 8:**
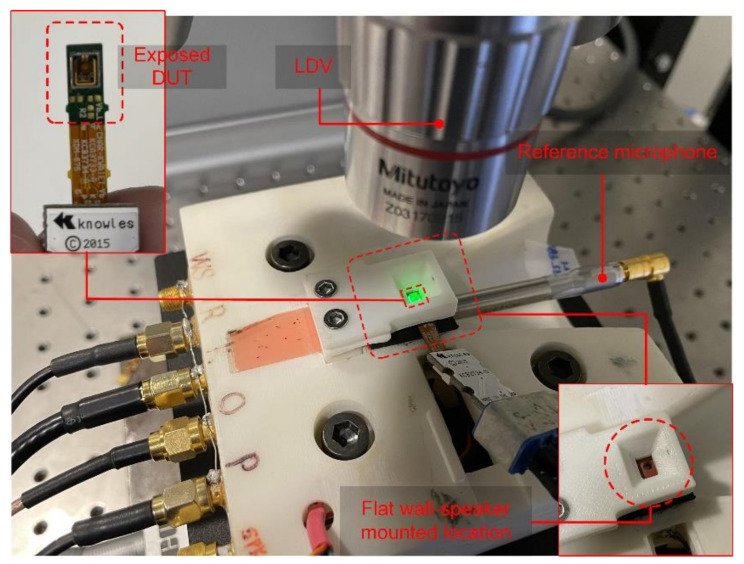
Experimental setup for the diaphragm compliance measurement using a scanning LDV.

The compliance of the diaphragm was measured here directly using a Laser Doppler Vibrometer (LDV). The device under test (DUT) is mounted on a pressure cavity, as shown in Figure 8. A small speaker provides an actuation pressure, while the LDV monitors the maximum diaphragm displacement. A reference microphone is used to calibrate the actuation signal to 1Pa. The resulting measurement gives the diaphragm compliance in units of m/Pa, corresponding to specific acoustic impedance. To convert to units of mechanical impedance, multiply by the diaphragm effective area, as shown in Table 5. 

**Figure 9:**
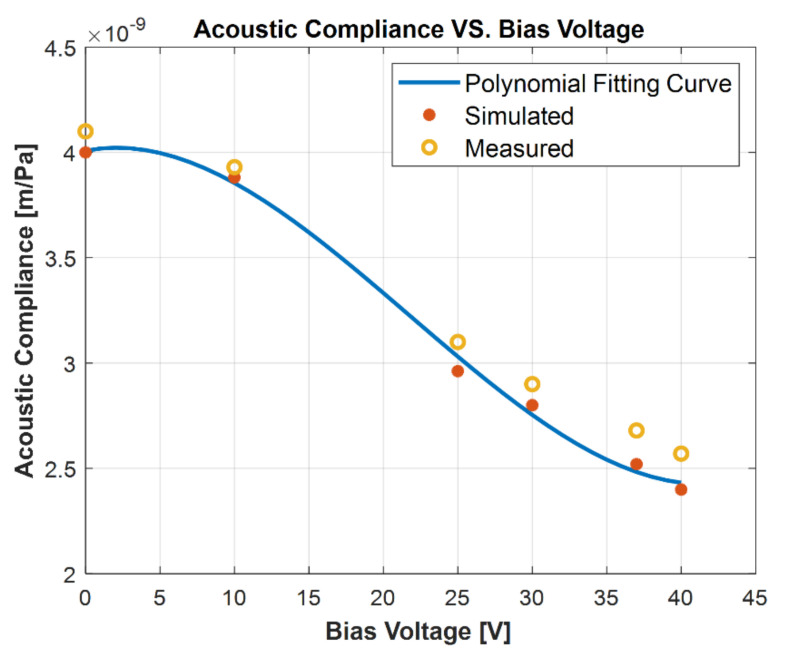
Measurement and simulation of the diaphragm acoustic compliance vs. bias voltage for the semiconstrained with peripheral post and center post boundary condition. The polynomial fitting curve is for the simulation.

An interesting observation was reported with the diaphragm acoustic compliance under bias voltage with the simply supported conditions using peripheral posts and center post design. The acoustic compliance decreased with increasing bias voltage in this configuration as opposed to increasing acoustic compliance with increasing bias voltage from electrostatic spring softening for constrained diaphragms. This particular phenomenon in the simply supported design is usually termed as the electrostatic spring stiffening effect. The apparent stiffening of the diaphragm is driven by the nonlinear static displacement of the diaphragm due to the electrostatic force. The FEA model is used to capture this behavior for the described model. The diaphragm acoustic compliance is plotted against the bias voltage in Figure 9.

## 5. Diaphragm FEA Model

FEA models have been developed to predict the mechanical response of the MEMS microphone diaphragm considering serpentine spring architecture with peripheral posts and a center post. To capture the fringe field effect due to the perforation holes on the backplate, a separate model was established that can be fed into the electromechanical model to simulate the coupled effect.

All FEA models are obtained through COMSOL Multiphysics^®^ software and a thermoviscous acoustic model was developed to calculate the acoustic damping of the MEMS.

### 5.1. Unit Cell Capacitance Model

**Figure 10:**
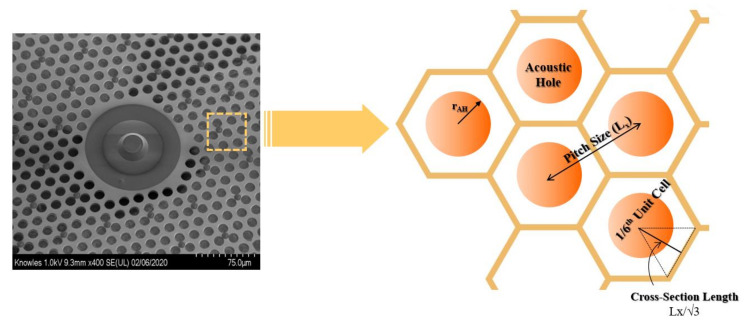
Hexagonal symmetry of the perforated backplate.

**Figure 11:**
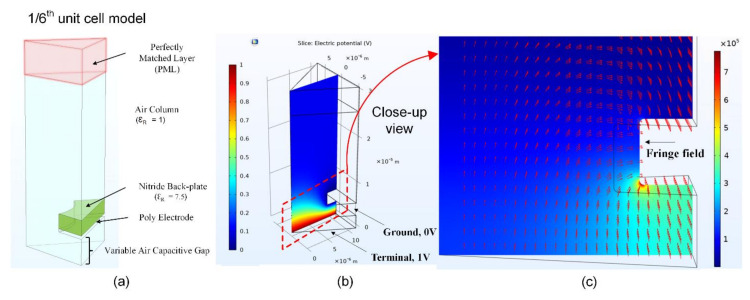
(**a**) Defined boundary condition for the 1/6th unit cell model with Si_3_N_4_ backplate and underlying polysilicon electrode. (**b**) Electric potential distribution with ground and terminal voltage. (**c**) Electric field distribution with fringing field effect.

We begin by calculating the correction factor of the electrostatic force and capacitance for the perforated backplate by taking advantage of the uniform hexagonal distribution across the electrode surface, as shown in Figure 10. For the unit cell model setup, we define the air gap and the perfectly matched layer (PML) on the top, as shown in Figure 11a. The permittivity of vacuum for air, Si_3_N_4_, and polysilicon materials is defined in the material properties section of the module. The electric potential of 1 V is applied to the electrode, and the ground is set to 0 V, as shown in Figure 11b. A fringing field effect can be captured using the 1/6th unit cell FEA. The fringe field distribution near the acoustic hole is as shown in Figure 11c.

**Figure 12:**
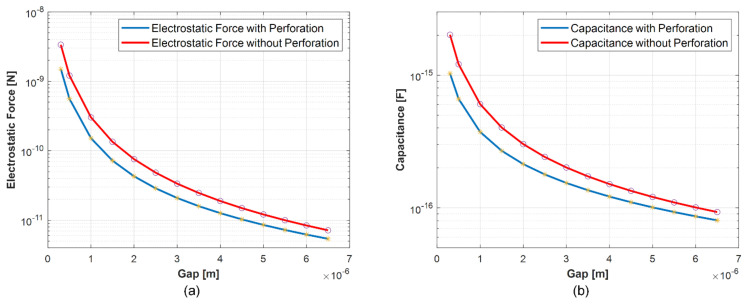
(**a**) Electrostatic force as a function of the gap between the electrodes for solid and perforated plates. (**b**) Capacitance as a function of the gap for solid and perforated plates.

The acoustic hole perforation ratio is approximately 54% of the defined electrode area. The electrostatic force and capacitance functions are shown in Figure 12a,b and are represented by fitting polynomials as a function of gap. These functions are used in the mechanical FEA model in the form of correction factors to calculate the motor capacitance and collapse voltage. For a gap of 5 µm, the force correction factor is determined to be 0.7, meaning 30% reduction in force with respect to the solid plate. Similarly, the capacitance correction factor is estimated to be 0.84, meaning 16% reduction in capacitance with respect to the solid plate.

### 5.2. Electromechanical Model

Symmetry of the diaphragm with a 45-degree segment is utilized to capture deflection, capacitance, and resonance modes under bias voltage. This model is obtained using COMSOL Multiphysics^®^ software with MEMS module. The electrostatic force *F* is fed into the model as per the coupling Equation (13), defined as

(13) $$F=\frac{\varepsilon AV^{2}}{2{\left(g_{0}-w\right)}^{2}}$$

where *A* is the surface area of the diaphragm, $g_{0}$ is the initial gap between the diaphragm and backplate electrode, and *w* is the diaphragm deflection under bias voltage, *V*. Furthermore, the above electrostatic force equation is multiplied by an analytical correction function to account for the effect of perforations. The material properties and dimensions of the polysilicon diaphragm are tabulated in Table 6, below.

**Figure 13:**
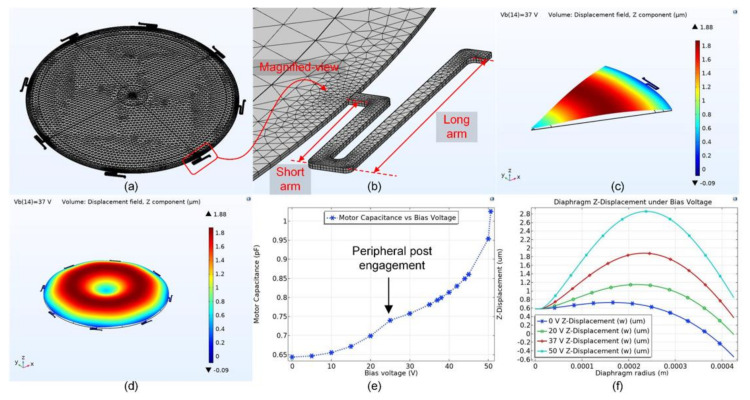
(**a**) Developed fine mesh for the full FEA model. (**b**) Developed mesh for the spring. (**c**) A 45-degree symmetry deflection model of the diaphragm under bias. (**d**) Full diaphragm deflection model showing a donut deflected shape under bias. (**e**) Capacitance as a function of bias voltage with collapse 50 V, parasitic capacitance not included. (**f**) Diaphragm deflection along the radius cross-section at different bias voltages.

The mesh and deflection shape of the diaphragm under bias voltage are shown in Figure 13a–d. Since there is a presence of a rigid center post on the backplate, the deflection shape of the diaphragm looks like that of a ‘donut’ under the bias voltage. The diaphragm capacitance is also plotted as a function of bias voltage until the diaphragm collapse happens with the backplate at 50 V. The presence of the small kink in Figure 13e at 25 V bias indicates engagement of the diaphragm with the peripheral posts. Figure 13f shows the deflected cross-section shape of the diaphragm under different bias voltages. At diaphragm radius *r* = 0 µm, the diaphragm is deflected to 0.6 µm at the center and is further restricted to move at center due to the presence of a rigid center post.

### 5.3. Resonance Modes

**Figure 14:**
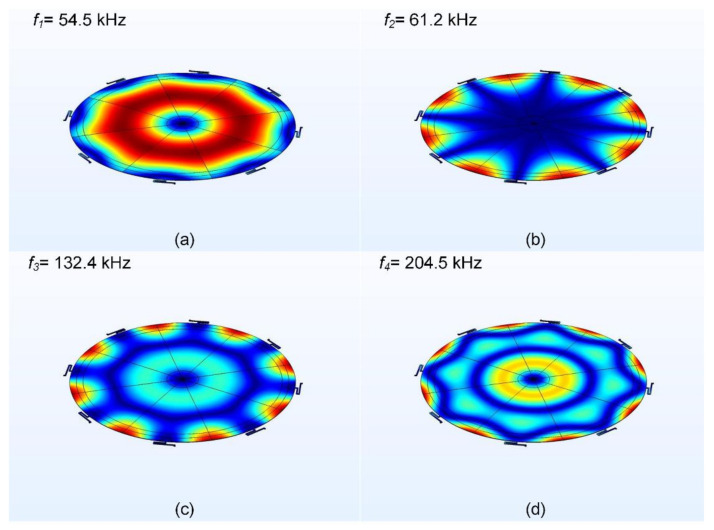
The first four vibration modes of the FEA model of the microphone diaphragm: (**a**) $f_{1}=54.5$ kHz, (**b**) $f_{2\;}=61.2$ kHz, (**c**) $f_{3}=132.4$ kHz, and (**d**) $f_{4}=204.5$ kHz. The red and blue surface profiles indicate regions of maximum and minimum deflections, respectively.

The resonance modes of the diaphragm under bias are extracted using the prestressed eigenfrequency study in COMSOL Multiphysics^®^. The first four vibration modes are as shown in Figure 14a–d. The first vibration mode is an ideal z-displacement vibration mode of the diaphragm. The first vibration mode (54.5 kHz) and the second mode (61.2 kHz) are high enough to not create any interference with the normal operating frequency range of microphone in the 20–20 kHz. The third and fourth vibration modes are even higher than 100 kHz. Note that the resonance obtained here is under vacuum condition, and no air damping has been used in the model. The structural diaphragm resonance under ambient pressure loading is discussed in Section 7.1, with the first resonance mode measured in air to be 39 kHz.

**Figure 15:**
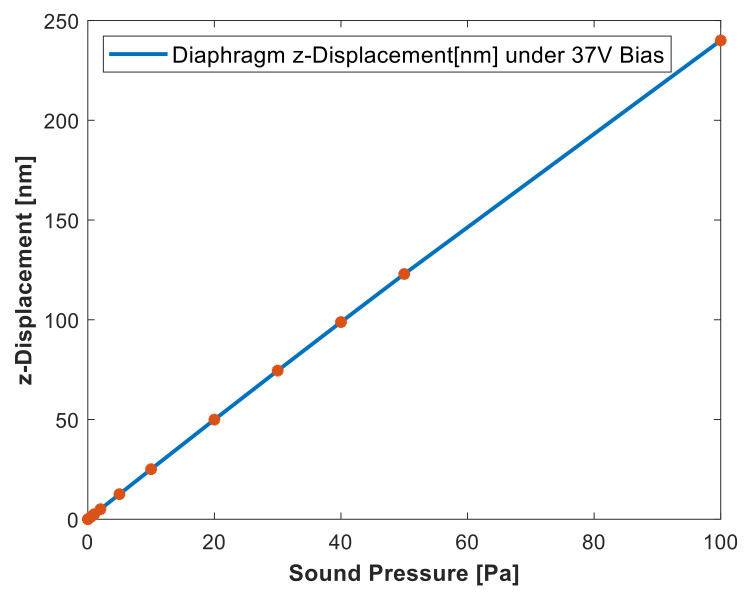
Simulation result of diaphragm deflection under the bias voltage as a function of sound pressure.

The deflection of the diaphragm under bias voltage is plotted against the applied sound pressure, as shown in Figure 15. For the small signal applied acoustic pressure, the diaphragm acoustic compliance can be calculated using the linear slope of the curve to be 2.5 nm/Pa. The acoustic compliance is the deflection averaged over the surface of the diaphragm under the applied sound pressure. The mechanical compliance of the diaphragm is calculated to be 7.154 $\times 10^{-3}$ m/N.

## 6. Noise Sources in MEMS Microphone

**Figure 16:**
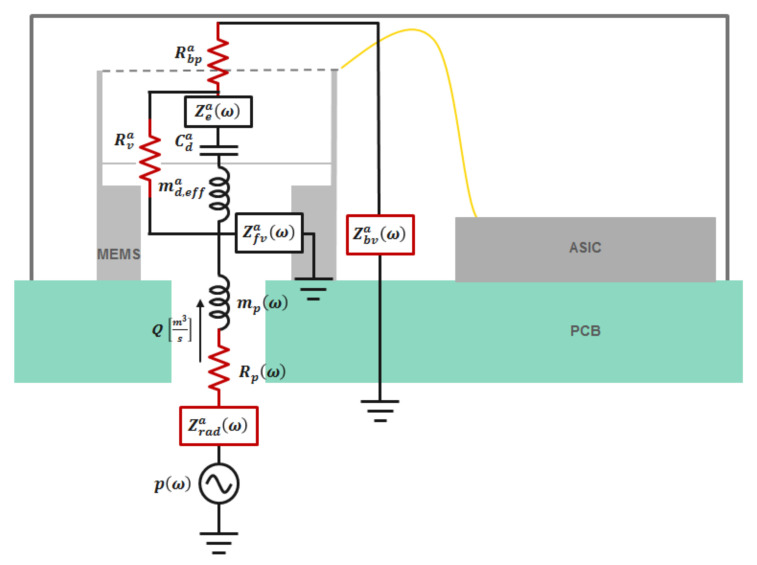
Simplified electroacoustic lumped model representing noise sources.

The four major acoustic sources of noise in MEMS microphones are acoustic port damping and vent resistance, which includes slit flow (around the perimeter of the diaphragm since it is not fully constrained) in parallel with flow through the pierce (hole punched on the diaphragm near the center post). The four major noise impedances are highlighted in red in the Figure 16. $Z^{a}{}_{rad},\;Z^{a}{}_{fv},\;Z^{a}{}_{e},\;$ and $Z^{a}{}_{bv}$ are the port, front volume, diaphragm, and back volume impedances, respectively. The analytical or FEA approach to obtain these parameters will be discussed in this section.

### 6.1. Analytical Calculation of Port and Cavity Acoustic Parameters

For the noise model, the dimensions and properties shown in Table 7 were used. 

The acoustical–domain lumped parameters for the acoustic port and cavities can be calculated using well-known analytical expressions, as shown in Table 8.

For calculating the port impedance, Beranek’s analytical equations are used [31]. 

**Figure 17:**
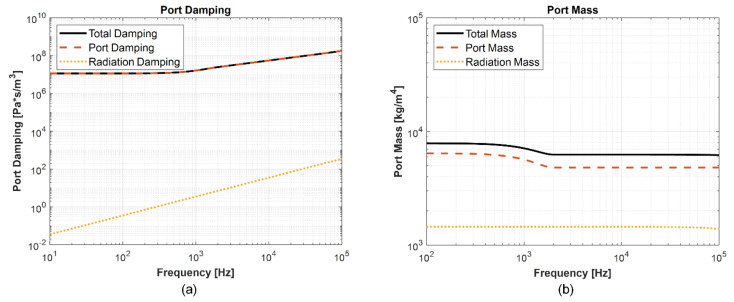
(**a**) Total damping obtained using port damping and radiation damping as function of frequency, and (**b**) total mass obtained using port mass and radiation mass as a function of frequency.

The radiation port impedance is given by Equation (14):

(14) $$Z_{rad}=\frac{\rho_{o}c}{\pi a_{p}^{2}}\left[\left(1-\frac{2J_{1}\left(2ka_{p}\right)}{2ka_{p}}\right)-j\frac{2H_{1}\left(2ka_{p}\right)}{2ka_{p}}\right]$$

where $H_{1}\left(z\right)\approx \frac{2}{\pi }-J_{0}\left(z\right)+\left(\frac{16}{\pi }-5\right)\frac{\sin z}{z}+\left(12-\frac{36}{\pi }\right)\frac{1-\cos z}{z^{2}}$, and $J_{1}\left(x\right)$ is the first-order Bessel function. For an approximation for the Sturve function of the first kind, $H_{1}\left(x\right)$ is used as given by [32]. The plots for port damping and port mass as a function of frequency, obtained using the lumped parameter model, are shown in Figure 17a,b.

### 6.2. Calculation of Enclosure Impedance

The thermal boundary layer thickness, $\delta_{t}$, as a function of frequency is given by

(15) $$\delta_{t}=\sqrt{\frac{2\kappa }{\omega \rho_{0}C_{p}}}$$

Equation (16) assumes the wall to be an isothermal boundary and ignores the influence of adjacent walls. The thermally corrected impedance for a thin parallelepiped enclosure with a height 2a is given by Equation (16) [33]:

(16) $$Z=\frac{1}{j\omega C_{a}\;\left[1+\left(\gamma -1\right)\left(\frac{\tanh\left(\beta a\right)}{\beta a}\right)\right]}$$

where $C_{a}$ is the adiabatic compliance of the air volume, *β* = $\sqrt{\frac{j\omega \rho_{0}C_{p}}{k}}$, $\rho_{0}$ is the density, k  is the thermal conductivity, γ is the ratio of specific heats for gas inside the enclosure, and $C_{p}$ is the specific heat at constant pressure of the gas inside the enclosure. For a more detailed discussion on this topic, see [33].

### 6.3. Vent Resistance

The LFRO of the microphone is set, effectively, by an RC high-pass filter formed between the total vent resistance, $R_{v,tot}\;$, and the back volume compliance, $C_{b,tot}$. Because the LFRO can be easily measured and the back volume compliance is generally well-known, the vent resistance can be calculated using Equation (17):

(17) $$R_{v,tot}\approx \frac{1}{2\pi f_{LFRO}C_{b,tot}}\;$$

### 6.4. Backplate Damping

Analytical solutions for the acoustic damping in perforated MEMS with a piston-like motion of the diaphragm and also a clamped circular diaphragm [34] have been developed. However, for the presented microphone, due to the complicated shape of the diaphragm under bias, developing a closed form analytical formula for the damping would be challenging. The damping of flexible structures can be accurately estimated with thermoviscous FEM simulations [35]. 

The Thermoviscous Acoustic (TA) module of the COMSOL Multiphysics^®^ software is used for three-dimensional FEM frequency domain simulations of the backplate acoustic damping. Due to the symmetric pattern of the posts, a 22.5 deg section of the plate is modeled, and the symmetry condition is applied to the edges. Appropriate no-slip and isothermal wall boundary conditions are applied to all pertinent included model surfaces. The deflected shape of the diaphragm under bias voltage from the diaphragm model in Section 5.2 is used for the static shape of the diaphragm in the damping model. The velocity of the diaphragm, as a function of the radius and angular coordinate of the diaphragm, is extracted from the diaphragm model. In the damping model, the diaphragm surface is driven with this velocity function.

A column of air and a PML are included at the top of the backplate to guarantee no reflection from the top. 

**Figure 18:**
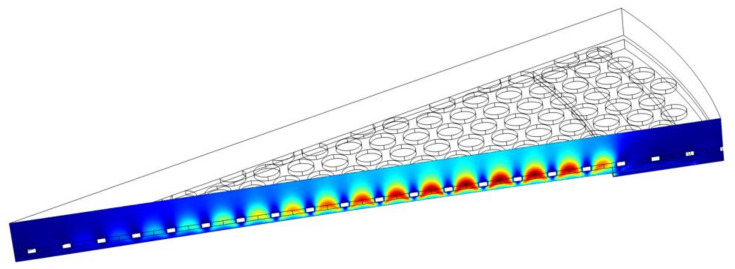
Cross-section of the velocity field in the FEM domain at 1 kHz. Red is associated with high velocity, and blue is associated with low velocity.

The cross-section of the velocity field from the FEM model is shown in Figure 18. The acoustic damping (resistance) is calculated from Equation (18):

(18) $$R_{bp}=\frac{P_{diss}}{\frac{1}{2}{\left|U\right|}^{2}}\;$$

where $P_{diss}$ is the total dissipated power calculated from volume integral of the total power dissipation density, and U is the diaphragm volume velocity. The simulated value for the damping was $R_{bp}=1.74\times 10^{8}\;\left(\mathrm{Pa}\cdot s/m^{3}\right)$.

## 7. Microphone Performance

### 7.1. Microphone Sensitivity

The sensitivity under bias voltage is defined by Equation (19):

(19) $$S=20\log_{10}\left(\frac{\Delta C\cdot V_{B}}{(C_{m}+C_{p}+C_{i})\cdot \Delta P}\right)$$

where $C_{m}$ is the diaphragm capacitance, $C_{p}$ is the parasitic capacitance, $C_{i}$ is the ASIC input capacitance of 0.215 pF, ΔC is the change in capacitance under the applied acoustic pressure, and ΔP is the pressure change accounting for the back volume compliance change due to diaphragm movement. 

**Figure 19:**
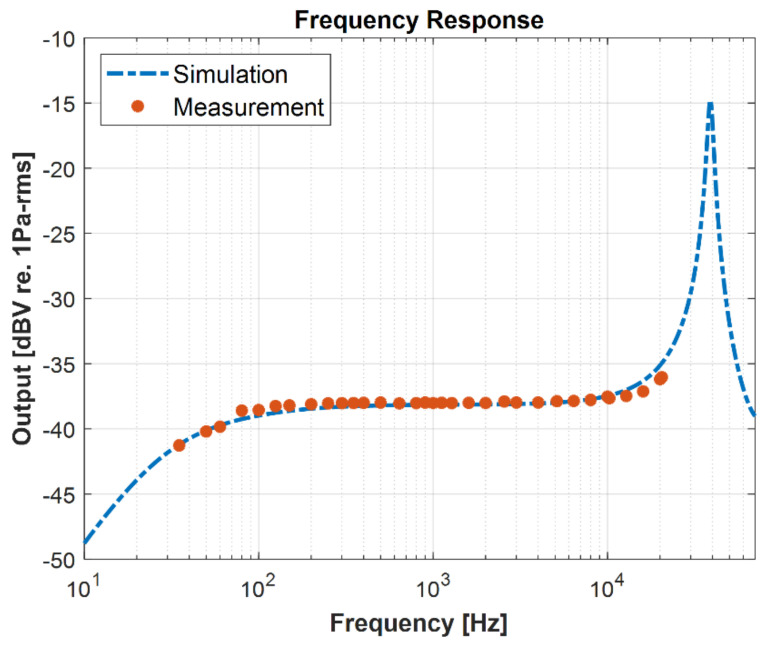
Measured frequency response along with the simulation.

The frequency response of the microphone was measured in an anechoic chamber and was compared to the simulated frequency response from the lumped element model shown in Figure 19. A good correlation between the measured and the simulated curves is established. The measured sensitivity at 1 kHz is −38 dBV/Pa. The microphone resonance in air is 39 kHz.

### 7.2. Noise

**Figure 20:**
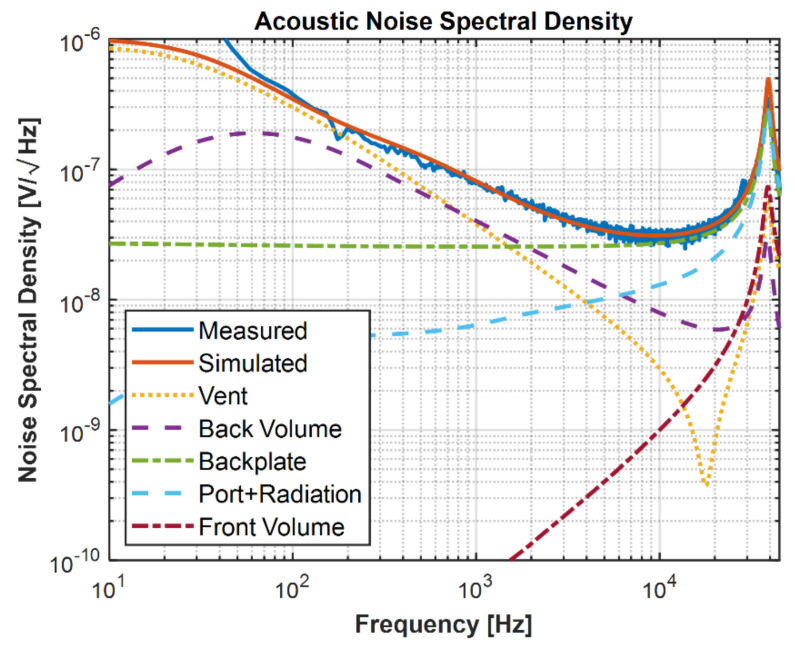
Measured and simulated noise spectral density. The component-wise noise spectra are plotted together to show their contributions to the simulated noise spectrum in total.

The noise spectrum of the microphone can be obtained by measuring the power spectral density in a sound isolation chamber. The measured noise spectral density is shown in Figure 20 with the simulation results from the lumped element model from Figure 16. Good agreement is observed between the simulated noise spectral density and the measured noise spectral density. The total noise spectrum is the incoherent superposition of all the noise sources. By solving the lumped circuit as per Figure 16 for responses from each noise source individually, the total noise can be calculated.

By subtracting the ASIC noise from the total microphone noise, the acoustic SNR was calculated to be 69 dBA. The acoustic SNR is a measure of the MEMS and package noise contributions, which is calculated by excluding the ASIC noise from the total noise. The typical A-weighted input-referred ASIC noise is approximately 3.4 µVrms. The microphone SNR obtained is 67 dBA in a 3.25 mm × 1.9 mm × 0.9 mm package.

**Figure 21:**
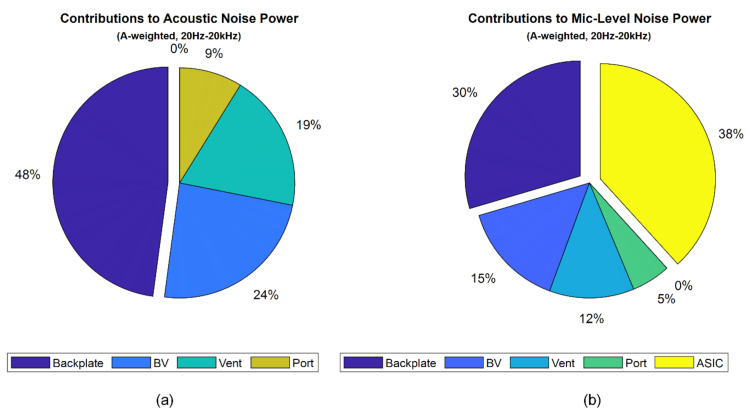
Pie charts for the total acoustic noise contributors from (**a**) MEMS only and (**b**) a package including MEMS and ASIC.

As shown in Figure 21a, the backplate damping with ~50% is the highest contributor for the acoustic noise source, followed by the back volume, vent, and port noises, respectively. At the microphone level, including ASIC, as shown in Figure 21b, the analog ASIC noise with 38% is the highest contributor to the noise source followed by backplate damping, back volume, vent, and port noises.

### 7.3. Resonance Peak

**Figure 22:**
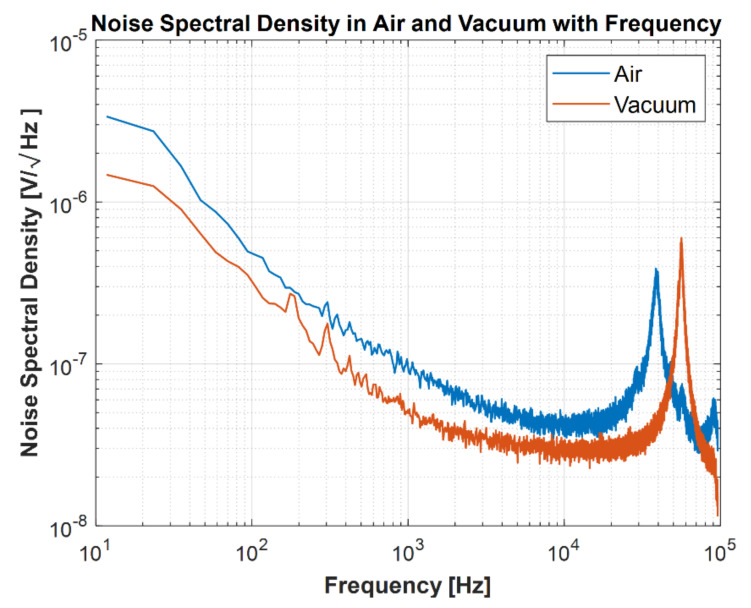
Noise spectral density in air and vacuum with respect to frequency.

The noise spectral density is plotted in Figure 22 against the frequency by measuring in vacuum and air. The resonance in the vacuum is purely the mechanical diaphragm resonance owing to no air damping and is reported to be 55.5 kHz, which is 2% higher than the predicted diaphragm resonance (54.5 kHz) using the FEA model in Figure 14. Due to the air loading effect, the microphone resonance in air is measured to be 39 kHz, which is in excellent agreement with the lumped element model prediction, as shown in Figure 20.

### 7.4. Total Harmonic Distortion

**Figure 23:**
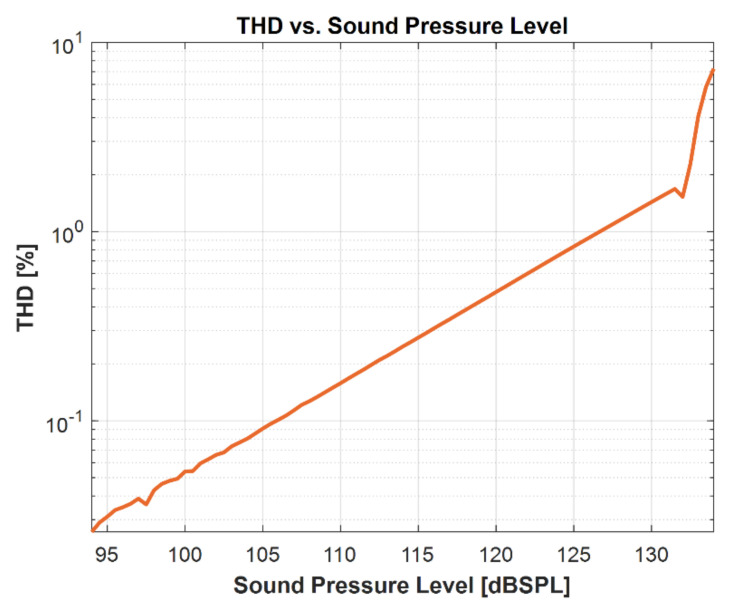
Measured THD curve.

Figure 23 shows the measured total harmonic distortion (THD) of the microphone, showing a distortion of 1.4% at 130 dB SPL and 7.2% at 134 dB SPL. The acoustic overload point (AOP) of 10% THD is higher than 134 dB SPL, at which point the electrical signal clips in the ASIC.

In Table 9, the electroacoustic performance of the proposed MEMS microphone is summarized. In addition, Table 10 shows a comparison with other bottom port MEMS microphones available in the market with a similar package size. Our proposed MEMS microphone compares favorably with the available microphones in the market in terms of sensitivity, SNR, and THD performance in a relatively smaller package.

## 8. Conclusions

A semiconstrained diaphragm with a unique spring design supported with peripheral posts and a center post has been developed with performance of 67 dBA SNR in a 3.25 mm × 1.9 mm × 0.9 mm package. This is among the highest SNR that has been reported in this package as compared to other commercially available microphones. The microphone has a diaphragm with eight serpentine springs consisting of a shorter arm connected to the diaphragm side and a longer arm constrained on the anchor finger. The peripheral posts near the springs have been designed along with a center support structure, providing a simply supported boundary condition for the diaphragm under bias voltage. An 85% increase in the effective area of the diaphragm in this configuration was found with respect to a constrained diaphragm and a 48% increase with respect to a simply supported diaphragm without the center post architecture. Under the bias condition, the effective area further increases by 15%, as compared to the unbiased case. Detailed analytical, FEA, and lumped element simulations were utilized to predict and optimize the performance levels of the microphone. The results of the FEA and lumped element simulations show a good agreement with the measurement values obtained for the microphone.

## Figures and Tables

**Table 1:** Description of domains in an acoustomechanical lumped model.

Domain	Flow Variable	Effort Variable	Impedance	Units of Impedance
Acoustical	volume velocity, U	pressure, P	$Z_{a}=\frac{P}{U}$	$\frac{Pa\cdot s}{m^{3}}\;\mathrm{or}\;\frac{N\cdot s}{m^{5}}\;$
Mechanical	velocity, v	force, F	$Z_{m}=\frac{F}{v}$	$\frac{N\cdot s}{m}$
Electrical	current, i	voltage, V	$Z_{e}=\frac{V}{i}$	Ω

**Table 2:** Lumped elements in a microphone model.

Element	Mechanical Equivalent Model	Electrical Equivalent Model	Acoustical Equivalent Model
Mass	$M_{m}$. (kg)	Inductance L (H)	$M_{a\;}=M_{m}/A_{d\_eff}{}^{2}$ (kg/m^4^)
Spring	$C_{m}$ (m/N)	Capacitance, C (F)	$C_{a}=C_{m}A_{d\_eff}{}^{2}$ (m^5^/N)
Damper	$B_{m}$ (N.s/m)	Resistance, R (Ω)	$R_{a\;}=B_{m}/A_{d\_eff}{}^{2}$ (N·s/m^5^)

**Table 3:** Description of the lumped elements used in Figure 4.

Category	Parameter	Description	Unit
Acoustical (Port and back cavity)	$R_{port}$	Acoustic port resistance	$N\cdot s/m^{5}$
	$L_{port}$	Acoustic port inductance	$N\cdot s^{2}/m^{5}$
	$L_{fvol}$	Front volume inductance	$N\cdot s^{2}/m^{5}$
	$C_{f}$	Front volume compliance	$m^{5}/N$
Acoustical (Vent)	$R_{vent}$	Vent flow resistance	$N\cdot s/m^{5}$
Acoustical (Backplate)	$R_{bph}$	Backplate hole resistance	$N\cdot s/m^{5}$
	$L_{bph}$	Backplate hole inductance	$N\cdot s^{2}/m^{5}$
Acoustical (Back cavity volume)	$C_{b}$	Back volume compliance	$m^{5}/N$
	$C_{rc1}$ $,\;C_{rc2}$ $,\;C_{rc3}$	TBL * compliance term #1, #2, and #3	$m^{5}/N$
	$R_{rc1}$ $,\;R_{rc2}$ $,\;R_{rc3}$	TBL damping term #1, #2, and #3	$N\cdot s/m^{5}$
Mechanical (Diaphragm)	$R_{dia}$	Diaphragm mechanical damper	N·s/m
	$L_{dia}$	Diaphragm mechanical mass	kg
	$C_{dia}$	Diaphragm mechanical compliance	mN
Mechanical (Backplate)	$R_{bp}$	Backplate mechanical damper	N·s/m
	$L_{bp}$	Backplate mechanical mass	kg
	$C_{bp}$	Backplate mechanical compliance	mN
Electrical (MEMS and ASIC)	$C_{MEMS}$	Motor Capacitance (including MEMS parasitic capacitances)	pF
	$C_{in}$	ASIC input Capacitance	pF

* TBL is abbreviated for thermal boundary layer.

**Table 4:** FEA simulated values of effective area coefficient for different diaphragm boundary conditions.

Diaphragm Boundary Condition	Volume Displacement, $\int_{0}^{a}y\left(r\right)2\pi rdr\;(m^{3})$	Maximum $\mathrm{Displacement},\;y_{c}$ (m)	Simulated Effective Area Coefficient, β
Constrained	$2.51\times 10^{-15}$	$1.33\;\times 10^{-8}$	0.33
Simply supported	$1.45\times 10^{-14}$	$5.57\times 10^{-8}$	0.46
Free plate with Peripheral Post	$1.01\times 10^{-14}$	$4.29\times 10^{-8}$	0.41
Free plate with Peripheral and Center Post	$1.33\times 10^{-15}$	$3.86\times 10^{-9}$	0.61
Semiconstrained with Peripheral Post	$9.22\times 10^{-15}$	$3.95\times 10^{-8}$	0.41
Semiconstrained with Peripheral Post and Center Post	$1.3\times 10^{-15}$	$3.74\times 10^{-9}$	0.61
Semiconstrained with Peripheral Post and Center Post under bias	$1\times 10^{-15}$	$2.52\times 10^{-9}$	0.7

**Table 5:** Summary of techniques for measuring diaphragm compliance.

Expression	Unit	Description
$C_{d,sa}=\frac{\delta_{max}}{p_{ref}}$	m/Pa	Specific Acoustic Impedance
$C_{d}=\frac{\delta_{max}}{p_{ref}A_{deff}}$	m/N	Mechanical Impedance

**Table 6:** Dimensions of the MEMS microphone diaphragm.

Property	Value
Diaphragm diameter	850 (μm)
Diaphragm thickness	1.4 (μm)
Spring long arm length	80 (μm)
Spring short arm length	45 (μm)
Spring width	8 (μm)
Gap between springs	4 (μm)
Spring count	8

**Table 7:** Microphone lumped parameter values.

Category	Parameter	Description	Value	Unit
Package	$V_{b}$	Back cavity volume	$1.676\times 10^{-9}$	$m^{3}$
	$l_{p}$	Acoustic port length	$250\;\times 10^{-6}$	m
	$a_{p}$	Acoustic port radius	$175\times 10^{-6}$	m
	$V_{f}$	Front cavity volume	$0.88\times 10^{-9}$	$m^{3}$
MEMS	$r_{d}$	Diaphragm radius	$425\times 10^{-6}$	m
	$t_{d}$	Diaphragm thickness	$1.4\times 10^{-6}$	m
	$r_{AH}$	Acoustic hole radius	$8.25\times 10^{-6}$	m
	$g_{o}$	Average gap after bias over electrode region	$4\times 10^{-6}$	m
	$V_{bias}$	Bias Voltage	37	V
	$C_{mems,tot}$	MEMS total capacitance	0.9	pF
	$C_{p}$	MEMS parasitic capacitance [30]	0.12	pF
	$\omega_{o,air}$	Diaphragm resonance in characterization package, measured in air	39	kHz
	$C_{d}^{a}$	Diaphragm compliance	2.5	nm/Pa
	$f_{c}$	Low-frequency corner	35	Hz
	$R_{bp}^{a}$	Backplate damping	$1.74\times 10^{8}$	$\mathrm{Pa}\cdot s/m^{3}$
	$m_{d\_eff}^{a}$	Effective acoustic diaphragm mass	$1.13\times 10^{-10}$	kg
	*α*	Effective mass coefficient for simply supported plate	0.296	dimensionless
	$A_{d\_eff}$	Effective diaphragm moving area	$3.52\times 10^{-7}$	$m^{2}$
	*β*	Effective area coefficient	0.7	dimensionless
	*φ*	Electrostatic coupling coefficient (Transduction factor)	$1.201\times 10^{5}$	V/N

**Table 8:** Calculation of port and cavity parameters.

Symbol	Description	Expression	Unit
$m_{p}$	Port mass	$m_{p}=\frac{\rho_{o}\left(l_{p}+1.7a_{p}\right)}{\pi a_{p}^{2}}$	$\frac{N\cdot s^{2}}{m^{5}}$
$R_{p}$	Port resistance	$R_{p}=\frac{8\eta l_{p}}{\pi a_{p}^{4}}$	$\frac{N\cdot s}{m^{5}}$
$C_{f}$	Front cavity compliance	$C_{f}=\frac{V_{f}}{\rho_{o}c^{2}}$	$m^{5}/N$
$C_{b}$	Back cavity compliance	$C_{b}=\frac{V_{b}}{\rho_{o}c^{2}}$	$m^{5}/N$

Where $l_{p}$ is the length of the acoustic port (m), $a_{p}$ is the radius of the acoustic port (m), $V_{f}$ is the volume of the front cavity (m^3^), and $V_{b}$ is the volume of the back cavity (m^3^). The physical constants represented here are density of air,$\;\rho_{0}$; viscosity of air, η; and speed of sound in air, c.

**Table 9:** Summary of the electroacoustic performance of the proposed MEMS microphone.

Property	Value
Sensitivity	−38 dBV/Pa
Signal-to-noise ratio (SNR)	67 dBA
Total harmonic distortion (THD)	7.2% at 134 dB SPL
Bandwidth	35–10 kHz
Motor capacitance	0.9 pF
Bias voltage	37 V
Pull-in voltage	50 V

**Table 10:** Performance comparison of the proposed design and commercially available MEMS microphones in market.

Microphone	ASIC Interface	Sensitivity	SNR (dBA)	AOP (dB SPL)	Package Size (mm × mm × mm)
Knowles, Proposed design	Analog	−38 dBV/Pa	67	>134	3.25 × 1.90 × 0.9
Infineon partner, MMA208-001 [36]	Analog	−38 dBV/Pa	67	135	3.35 × 2.50 × 0.98
Infineon partner, MMA208-W02 [36]	Analog	−38 dBV/Pa	66	136	3.35 × 2.50 × 0.98
Infineon partner, MA-ERA381-H43-1 [36]	Analog	−38 dBV/Pa	65.5	137	3.35 × 2.50 × 0.98
Infineon partner, S14OB381 [36]	Analog	−38 dBV/Pa	65	135	3.35 × 2.50 × 0.98
TDK InvenSense, ICS-40618 [37]	Analog	−38 dBV/Pa	67	132	3.5 × 2.65 × 0.98
ZillTek, ZTS6554 [38]	Analog	−37 dBV/Pa	67	120	3.35 × 2.50 × 0.98
ZillTek, ZTS6054 [38]	Analog	−38 dBV/Pa	65	125	3.35 × 2.50 × 0.98
TDK InvenSense, ICS-4078 [39]	Analog	−38 dBV/Pa	66	135	3.35 × 2.50 × 0.98
